# Quality of life changes after electrochemotherapy: a prospective single-center analysis

**DOI:** 10.1038/s41598-025-00782-0

**Published:** 2025-05-09

**Authors:** Petra Rózsa, Ferenc Rárosi, Henriette Ócsai, Eszter Baltás, Judit Oláh, Lajos Kemény, Rolland Gyulai, Erika Gabriella Kis

**Affiliations:** 1 Department of Dermatology and Allergology, Albert Szent-Györgyi Medical School, University of Szeged, Korányi fasor 6, Szeged, 6720 Hungary; 2 Department of Medical Physics and Medical Informatics, Albert Szent-Györgyi Medical School, University of Szeged, Korányi fasor 9, Szeged, 6720 Hungary; 3 HUN-REN-SZTE Dermatological Research Group, Szeged, 6720 Hungary; 4 Department of Dermatology, Venereology and Oncodermatology, Medical School, University of Pécs, Pécs, Hungary; 5 Department of Oncotherapy, Albert Szent-Györgyi Medical School, University of Szeged, Szeged, Hungary

**Keywords:** Electrochemotherapy, Quality of life, Skin cancer, Surgical oncology, Skin cancer, Quality of life

## Abstract

The rising prevalence of cutaneous and subcutaneous tumors has driven interest in electrochemotherapy (ECT) as a potential treatment. However, patient-reported outcomes remain underexplored. This study aims to assess the short-term impact of ECT on the quality of life (QoL) of patients, addressing a gap in the current literature. A prospective study evaluated 62 patients treated with ECT between 2015 and 2022. QoL was measured using the EQ-5D-3L questionnaire, calculating EQ-5D-index and assessing health state (EQ-VAS) and pain (pain-VAS). Subgroup analysis was conducted based on tumor histology, previous radiotherapy, and tumor size. Statistical analysis was performed using SPSS 29.0.0. The median age was 70 years, with a median follow-up of 47 days. Pre-treatment, 38.7% of patients reported pain/discomfort, and 24% had anxiety/depression. Post-treatment, these decreased to 32.2% and 19%, respectively. While the EQ-VAS and EQ-5D-3L scores showed a non-significant increase, pain-VAS decreased. Significant improvements were seen in patients with previous radiotherapy (EQ-VAS, *p* = 0.047; EQ-5D-index, *p* = 0.012) and smaller tumors (EQ-VAS, *p* = 0.035; pain-VAS, *p* = 0.029). ECT demonstrates a significant short-term benefit in maintaining or improving QoL in patients with cutaneous malignancies.

## Introduction

Electrochemotherapy (ECT) is a well-established method for treating cutaneous and subcutaneous tumors^1,2^. Initially employed predominantly for palliative purposes, it has evolved into a preferred curative treatment for patients with melanoma in-transit metastases and those with keratinocyte cancers (KC) in certain cases.

**Fig. 1:**
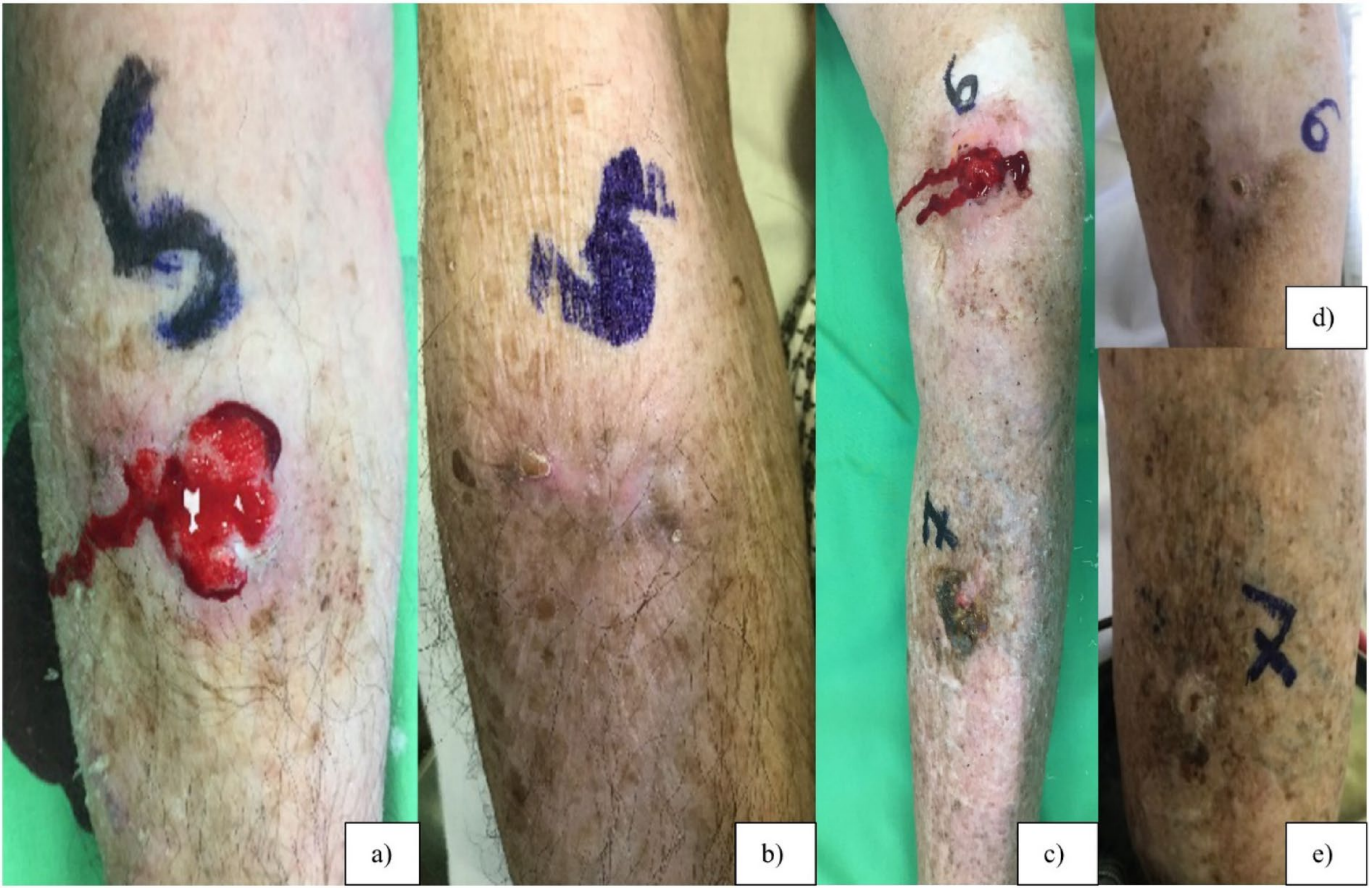
This 86-year-old patient had bleeding, ulcerating, painful and odorous tumor nodules on his lower limbs (**a**,** c**). We treated 16 tumor nodules with ECT, which resulted with a huge improvement in his QoL, as the symptomatic tumor nodules were no longer present at the 7 months follow-up (**b**, **d**, **e**).

ECT operates on the principle of reversible electroporation, serving as a physical drug-delivery system to facilitate the entry of non-permeant or poorly permeable molecules into cells. Short electric pulses locally applied induce an increase in membrane permeability, allowing the penetration of chemotherapeutic drug molecules. This technique combines electroporation with chemotherapeutic drugs, with bleomycin and cisplatin identified as the most efficient agents in conjunction with electroporation, exhibiting increased cytotoxicity by 8000 and 80-fold, respectively^3^Noteworthy advantages include cell-type selectivity, sparing healthy cells while targeting rapidly dividing tumor cells, thereby enabling treatment with a wide safety margin. ECT is compatible with previously irradiated or operated areas and can be seamlessly integrated with other local or systemic therapies, such as radiation or immunotherapy, with the option for repetition if necessary.An additional favorable aspect of ECT is its vascular effect: it induces a “vascular lock” through vasoconstriction at the site of the electric pulse application. This effect negatively affects neovascularization, particularly affecting rapidly dividing endothelial cells similar to tumor cells^4^ It is particularly advantageous when treating bleeding tumor nodules (Fig. 1).

ECT can be used to treat cutaneous or subcutaneous tumor nodules regardless of their histological types. In patients with numerous KCs, particularly those located in the head and neck area., it can be used with a curative intent. In such instances, ECT offers the potential of comparable tumor control to surgical excision and better cosmetic outcomes^5^ In melanoma patients with in-transit metastases, the goal of the treatment is to offer local tumor control.In the context of unresectable, locally invasive and/or destructive tumors (either primary or secondary), ECT serves a palliative role, providing relief for the patients with large, symptomatic cutaneous tumors.

The advent of advanced systemic oncological treatments like immunotherapy and checkpoint inhibitors has led to increased patient survival. Consequently, individuals now must cope with cutaneous malignancies for an extended duration. In managing cutaneous metastases, the objective is to mitigate symptoms such as pain, odor, ulceration, suppuration, or bleeding. These conditions significantly impair a patient’s quality of life.

The purpose of this study was to evaluate the effect of ECT treatment on the QoL and pain levels of our patients. We aimed to investigate, whether differences in the histological type of the tumors, previous irradiation or tumor size had any impact on the QoL changes.

## Methods

### Study summary

A prospective, observational, single-center study was carried out to evaluate the impact of ECT on the patients’ QoL. All patients gave written informed consent (ethical approval: ECT-REPRO-002, 9/2016-SZTE).

Data collection was carried out on patient demographics, medical history, nodule sizes and localization, details of treatment and QoL. Patients with primary or secondary cutaenous tumors were included in the study after the decision of our multidisciplinary tumor board according to the ESOPE criteria.

At the baseline visit before the treatment (1 day before or on the day of the treatment), data were collected about patients’ demographics, primary tumor, disease stage, previous local and systemic treatments. QoL and pain questionnaires were also completed before the treatment.

ECT sessions were carried out according to SOP. The treatment was performed under local or general anaesthesia. Bleomycin was administered either intravenously in a bolus injection at a dose of 15,000 IU/m^2^ body surface area, or directly into the tumor lesions. In the latter case, the dose was 1000 IU/ml considering the tumor volume. Electric pulses were generated by Cliniporator Pulse Generator (IGEA, s.r.l., Carpi, Italy), and we used fixed geometry, standard electrodes (hexagonal or row needle electrodes).

After treatment, the follow-up visit was scheduled in 1–3 months, where the patients were asked to fill out the questionnaires. The median follow-up time was 47 days (28–91).

### Questionnaires

**Fig. 2:**
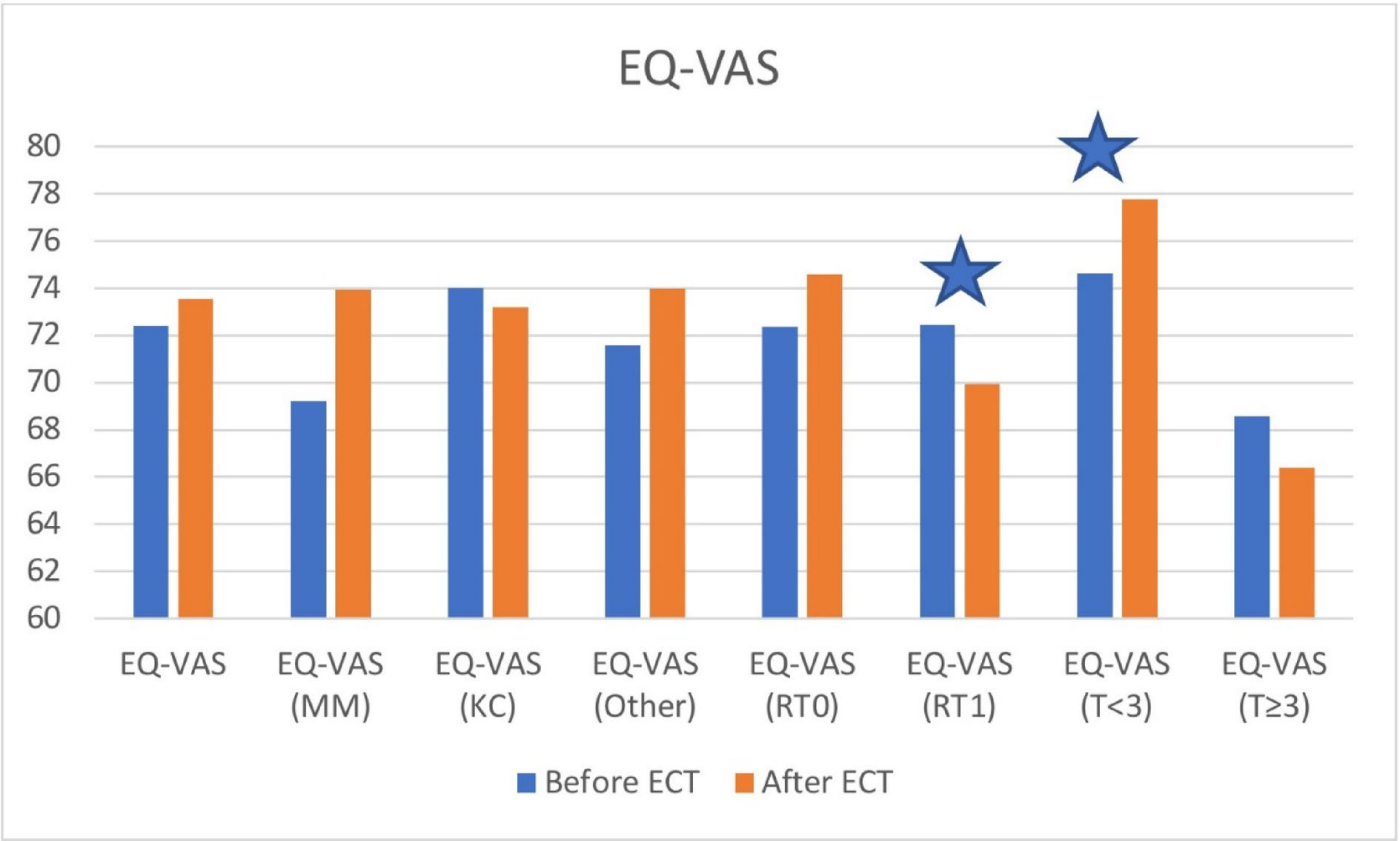
EQ-VAS values before and after treatment including subgroup analysis. An asterisk is showing statistically significant difference.

For the evaluation of the the QoL and pain experienced by the patients, standardized, internationally accepted, validated questionnaires were used. The EQ-5D-3L questionnaire includes the self-assessment of the following dimensions: mobility, self-care, usual activities, pain/discomfort, anxiety/depression, each dimension has 3 levels (Fig. 2). The patient was asked to choose from 3 levels, which indicate: no problem/some problem/extreme problem in every dimension. The general health state and pain were evaluated on a visual analogue scale: EQ-VAS (0-100) and Pain-VAS (0–10). Mild pain is considered to be a VAS score between 0 and 2, moderate pain is a score between 3 and 4, severe pain is 5 or above.

The EQ-5D-index (0–1) is a summary index calculated using a formula that assigns weights to each level of the 5 dimensions of the questionnaire, where ’1’ is the value of complete health. The value sets are created with the use of a representative sample of the general population in a specific country or region, where participants are asked to assign values to EQ-5D health states, so this method is standardized. In our study, we used the European value set.

These questionnaires were completed in the context of a face-to-face interview with the patients. They are easy to understand, it takes only a couple of minutes to complete them, and they are descriptive of the patients’ QoL. Each questionnaire was evaluated before treatment, which gave us a baseline value. After the ECT session, the questionnaires are repeated at every follow-up visit, so that we can analyse the changes in the patients’ QoL and pain values.

### Statistical analysis

Categorical data were expressed as frequencies and relative frequencies (percentages), continuous data were expressed as mean ± standard deviation. Data were split into subgroups according to three variables: histological types of tumors, previous radiotherapy, and tumor size. The values of the EQ-VAS, Pain-VAS and EQ-5D-index before and after ECT treatment were compared with paired t-test. Differences in EQ-VAS, Pain-VAS, and EQ-5D index scores among patient subgroups defined by tumor localization and comorbidity categories were analyzed using one-way ANOVA. P-values less than 0.05 were considered as statistically significant. IBM SPSS software (Statistical Packages for Social Sciences) 29.0.0.0 (version 241) software was used for the statistical analysis.

## Results

### Patients

Our study included 62 patients (42 male and 20 female) who underwent ECT between 2015.10.22. and 2022.06.21 (Table 1). The median age was 70 years (12–91 years). All patients had an ECOG status of 0 or 1. The three subtypes according to histology were the following: 14 patients (23%) had metastases of malignant melanoma (MM), 40 (56%) had keratinocyte cancer (KC: basal cell carcinoma (BCC) or squamous cell carcinoma (SCC)) and 13 patients (21%) had other cutaneous tumors (metastases of breast ductal adenocarcinoma, Kaposi’s sarcoma, angiosarcoma, epitheloid sarcoma, malignant Schwannoma). 39 patients (47%) had tumor nodules smaller than 3 centimeters (T < 3), and 14 patients (28%) had previously received radiotherapy (RT1). The number of tumors treated per patient ranged from 1 to 33 (median: 6).

**Table 1:** Patient and tumor characteristics.

Gender		
Male	42	68%
Female	20	32%
Age (years)		
Median	70	
Range	12–91	
Diagnosis		
Metastasis of malignant melanoma (MM)	14	23%
Keratinocyte carcinoma (KC)	35	56%
Others^a^	13	21%
Previous irradiation		
Yes (RT1)	14	28%
No (RT0)	48	72%
Tumor size		
Patients with no lesion > 3 cm (T < 3)	39	47%
Patients with at least one lesion ≥ 3 cm (T ≥ 3)	23	53%

^a^Metastasis of breast ductal adenocarcinoma, Kaposi’s sarcoma, angiosarcoma, epitheloid sarcoma, malignant Schwannoma.

Patients were also categorized based on the localization of the treated tumors into groups involving the head and neck region (*n* = 20), limbs (*n* = 14), trunk (*n* = 4), or multiple sites (*n* = 24).Additionally, they were grouped based on the number of comorbidities: those with no comorbidities (*n* = 6), those with 1–2 comorbidities (*n* = 29), and those with 3 or more (*n* = 27).

### EQ-5D-3L questionnaire

The number and percentage of patients who reported no or some problems in each dimension of the EQ-5D-3L can be seen in Table 2. Since we only included patients, whose ECOG status was 0 or 1, as it was expected, at the baseline, most of our patients had no problem with mobility (97%), self-care (98%) or usual activities (100%). After the treatment, these numbers didn’t change significantly.

The percentage of patients having no pain/discomfort increased from 61,3 to 67,7% after the treatment. Regarding anxiety and depression, 24% of patients reported some issues, which decreased to 19% after ECT.

**Table 2:** Numbers and proportions of patients reporting EQ-5D levels before and after ECT.

	Mobility		Self-care		Usual activities		Pain/discomfort		Anxiety/depression	
	Pre	Post	Pre	Post	Pre	Post	Pre	Post	Pre	Post
Level 1	60 (97%)	59 (95%)	61 (98%)	62 (100%)	62 (100%)	61 (98%)	38 (61,3%)	42 (67,7%)	47 (76%)	50 (81%)
Level 2	2 (3%)	3 (5%)	1 (2%)	0	0	1 (2%)	17 (27,4%)	14 (22,6%)	13 (21%)	11 (17%)
Level 3	0	0	0	0	0	0	7 (11,3%)	6 (9,7%)	2 (3%)	1 (2%)
Reporting some problems	2 (3%)	3 (5%)	1 (2%)	0	0	1 (2%)	24 (38,7%)	20 (32,3%)	15 (24%)	12 (19%)

### EQ-VAS

EQ-VAS, Pain-VAS and EQ-5D-index values before and after ECT are shown in Table 3. EQ-VAS values are shown in Fig. 2. Before the ECT session, the mean EQ-VAS reported by our patients was 72.39 ± 13.201, which increased to 73.55 ± 15.696 three months later. Although the increase was not statistically significant (*p* = 0.438), this is still a favorable outcome considering the treatment was carried out with a palliative intention in most of the cases and we could preserve the patients’ overall health state.

**Table 3:** EQ-VAS, Pain-VAS and EQ-5D-index values before and after ECT including subgroup analysis.

	Before ECT	After ECT	
EQ-VAS	72.39 ± 13.201	73.55 ± 15.696	*p* = 0.438
EQ-VAS (MM)	69.21 ± 17.979	73.93 ± 16.193	*p* = 0.079
EQ-VAS (KC)	74.03 ± 11.637	73.21 ± 17.112	*p* = 0.728
EQ-VAS (Other)	71.57 ± 11.501	74.00 ± 12.235	*p* = 0.257
EQ-VAS (RT0)	72.38 ± 14.002	74.60 ± 16.706	*p* = 0.240
EQ-VAS (RT1)	72.43 ± 10.435	69.93 ± 11.34	*p* = 0.047
EQ-VAS (T < 3)	74.64 ± 13.144	77.77 ± 12.274	*p* = 0.035
EQ-VAS (T ≥ 3)	68.57 ± 12.67	66.39 ± 18.377	*p* = 0.494
Pain-VAS	1.89 ± 2.765	1.68 ± 2.715	*p* = 0.287
Pain-VAS (MM)	2.21 ± 2.607	2.43 ± 3.368	*p* = 0.671
Pain-VAS (KC)	1.76 ± 3.066	1.32 ± 2.579	*p* = 0.096
Pain-VAS (Other)	1.86 ± 2.248	1.79 ± 2.326	*p* = 0.836
Pain-VAS (RT0)	1.69 ± 2.845	1.50 ± 2.806	*p* = 0.430
Pain-VAS (RT1)	2.57 ± 2.441	2.29 ± 2.367	*p* = 0.391
Pain-VAS (T < 3)	1.54 ± 2.634	1.03 ± 2.218	*p* = 0.029
Pain-VAS (T ≥ 3)	2.48 ± 2.937	2.78 ± 3.147	*p* = 0.382
EQ-5D-index	0.8044 ± 0.2297	0.8447 ± 0.2208	*p* = 0.132
EQ-5D-index (MM)	0.8163 ± 0.0622	0.7539 ± 0.0693	*p* = 0.279
EQ-5D-index (KC)	0.8147 ± 0.0397	0.8831 ± 0.0352	*p* = 0.065
EQ-5D-index (Other)	0.7674 ± 0.0632	0.8422 ± 0.0546	*p* = 0.157
EQ-5D-index (RT0)	0.8424 ± 0.2225	0.8488 ± 0.2254	*p* = 0.827
EQ-5D-index (RT1)	0.674 ± 0.2122	0.8309 ± 0.2116	*p* = 0.012
EQ-5D-index (T < 3)	0.8098 ± 0.2265	0.8889 ± 0.1925	*p* = 0.012
EQ-5D-index (T ≥ 3)	0.7952 ± 0.2401	0.7698 ± 0.2486	*p* = 0.602

There was no statistically significant difference in the changes in EQ-VAS values between the subgroups divided by histological types, tumor localisation and comorbidites.

Patients with previously irradiated tumor nodules had a significantly lower EQ-VAS score after the treatment (*p* = 0.047), while the other group had a statistically not significant increase (*p* = 0.240). Tumor size also had an effect on changes in the EQ-VAS. Those patients, who had smaller tumors, their EQ-VAS was significantly higher after the treatment (*p* = 0.035). Patients with bigger tumor nodules had a slight decrease in this value.

### Pain-VAS

**Fig. 3:**
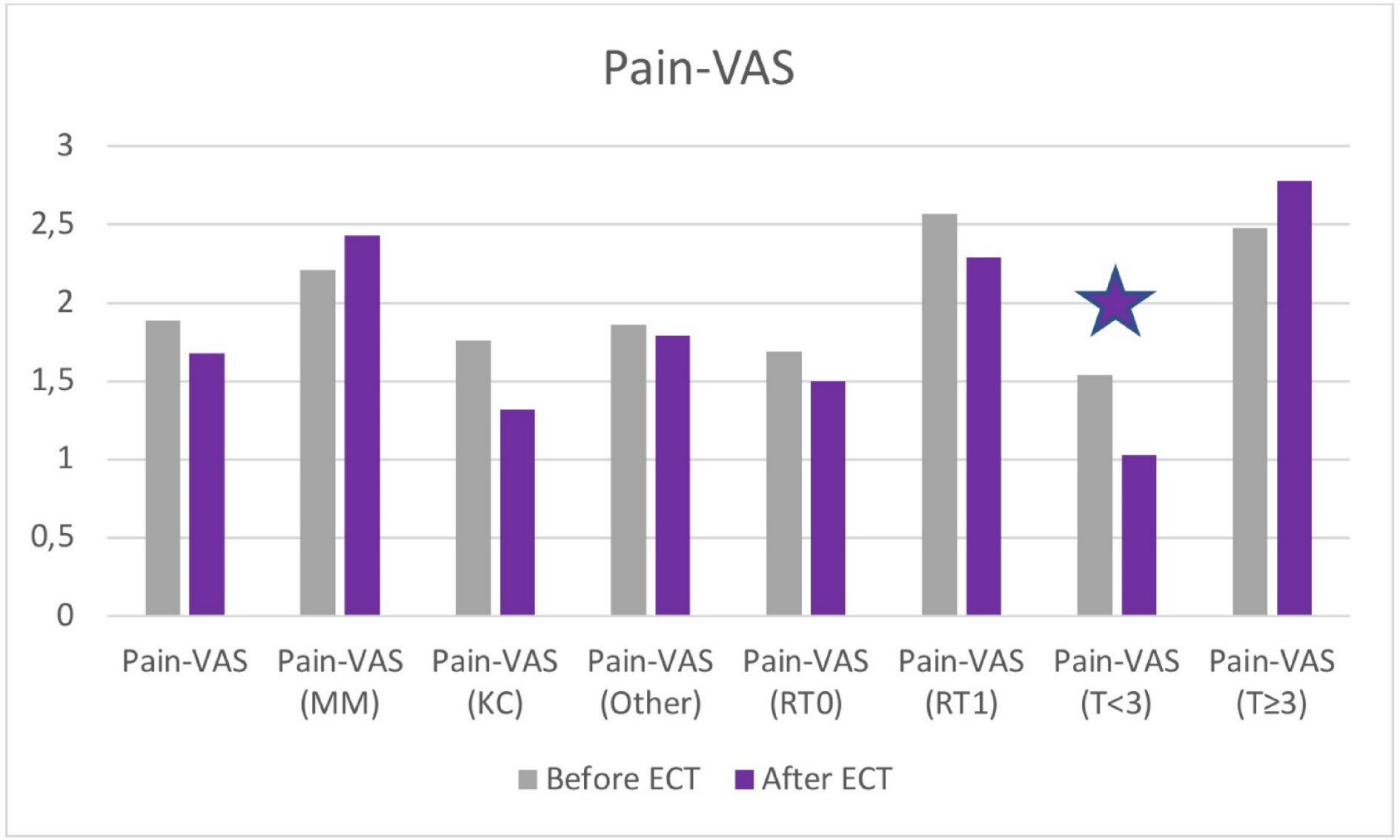
Pain-VAS values before and after treatment including subgroup analysis. An asterisk is showing statistically significant difference.

Pain-VAS values are shown in Fig. 3. The pain value of the patients decreased from 1.89 ± 2.765 to 1.68 ± 2.715 months after ECT (*p* = 0.287). There was no difference observed according to tumor histology, previous irradiation, tumor localisation or comorbidites. Patients with smaller tumor nodules had a significantly lower level of pain after the treatment (*p* = 0.029). As for those patients, who had at least one tumor bigger than 3 cm, a minimal increase was observed in the pain scores. Aside from pain, only temporary and localized side effects were noted, such as erythema, edema, hyperpigmentation, and transient ulceration in some cases.

### EQ-5D index value

**Fig. 4:**
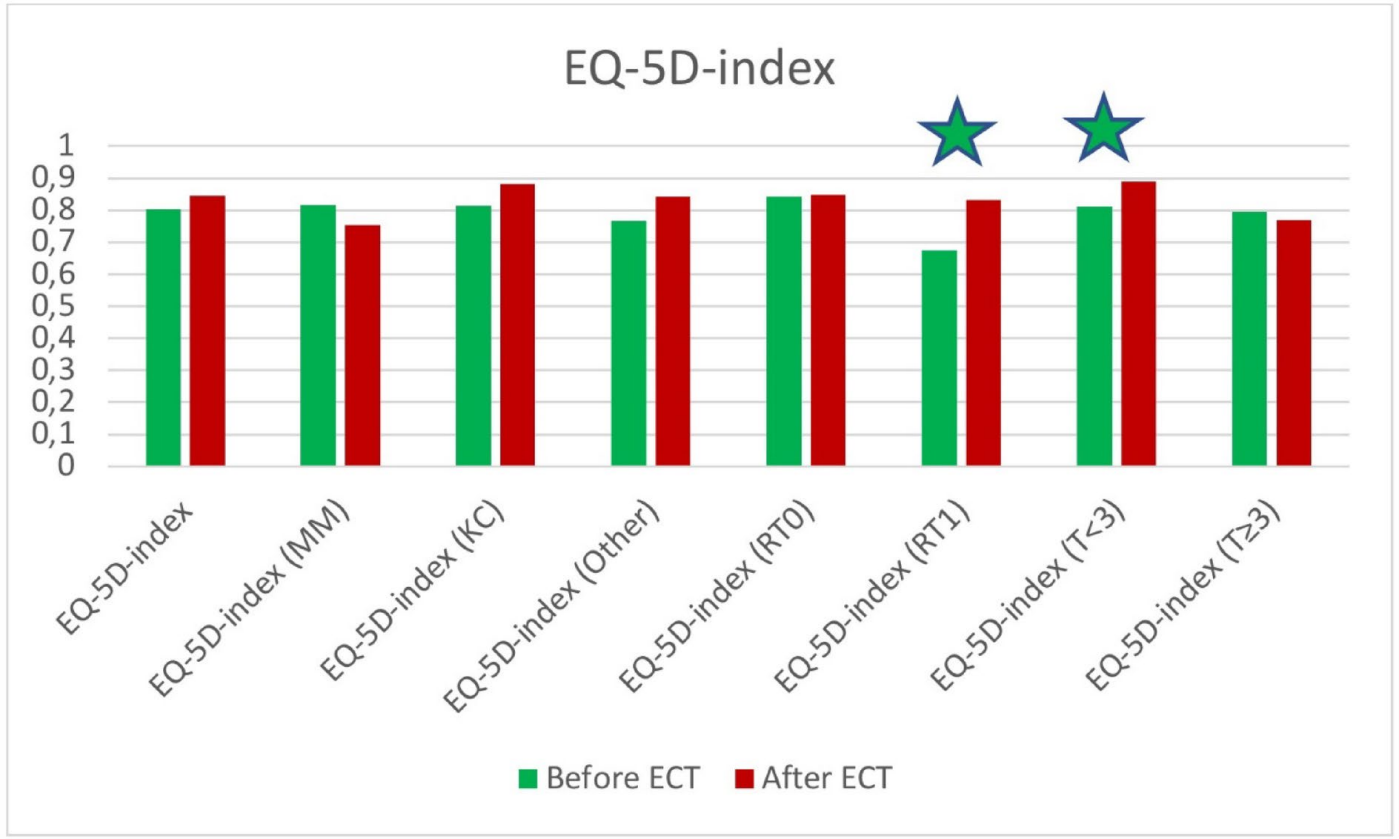
EQ-5D index values before and after treatment including subgroup analysis. An asterisk is showing statistically significant difference.

The EQ-5D index values are shown in Fig. 4. The average value was 0.8044 ± 0.2297 before ECT and 0.8447 ± 0.2208 after, so the increase is not statistically significant (*p* = 0.132). There were no statistically significant differences between the EQ-5D index values in the different subgroups divided according to tumor histology, localisation and comorbidites. Patients who had previously received radiotherapy presented with a significant increase in the EQ-5D index values (*p* = 0.012). When assessing the index values of those with smaller diameter tumors, they had a significant increase (*p* = 0.012), those with larger lesions, they had a nonsignificant decrease.

## Discussion

Patient-reported quality of life is a key outcome, particularly in oncology. Whether the treatment aim is curative or palliative, maintaining or improving quality of life is crucial. Primary or secondary cutaneous/subcutaneous tumors are often painful, odorous, and prone to ulceration and infection. They can also cause significant disfigurement, making them physically and emotionally distressing, while it can be challenging to manage them effectively. However, while clinical outcomes such as tumor response and survival rates have been well-documented, far less attention has been given to patient-reported outcomes (PROs), particularly the impact on QoL. In 2020., Haslam et al. published an article, in which they reviewed 149 oncological studies^6^ They aimed to determine whether the QoL assessment was conducted at various stages, including during treatment, shortly afterward, during follow-up, and until the time of death. Their findings indicated that the majority of studies did not include assessments of QoL.

The number of indications for which ECT is successfully used is rapidly increasing. Its effectiveness has already been proven by clinical trials, and it is recommended by several international guidelines. In a large-scale study, the authors reported that among 2,482 tumor lesions, the overall response (OR) rate was 85%^7^. In 2021, Perrone et al. conducted a multicenter prospective observational study on patients with vulvar carcinoma treated with ECT^8^ The EQ-5D-5L scores remained stable, but pain (VAS) and FACT-V scores improved statistically significantly at early and late follow-up (2 and 4 months, respectively). They also did a subgroup analysis and found that those who had stable or progressive disease, posterior site and either multiple or larger than 3 cm tumor nodules, they had worse QoL.

In 2021 Riva et al. published an article focusing on the quality of life of patients with cutaneous or mucosal head and neck tumors treated with ECT^9^ They used the EORTC QLQ-C30 and EORTC QLQ-H&N35 questionnaires on 27 patients, and found a significant increase in global health status, social functioning, and a significant decrease in pain, and appetite loss, and they managed to stop bleeding in 7/7 of their patients. The other parameters remained stable after the ECT.

In 2022, Campana et al. evaluated changes in the EQ-5D and EQ-VAS scores using the minimally important difference (MID) cut-offs^10^ The MID is defined as the smallest change in a PRO measure that patients perceive as beneficial or that would prompt a change in treatment^11^. They found that among the 378 melanoma patients treated with ECT, both scores stayed within the MID range – especially in complete responders. They conducted an analysis at five different time points and found that there was a transient decrease in the dimensions of pain/discomfort and mobility, and a persistent decline in self-care and usual activities.

The International Network for Sharing Practices of ECT (INSPECT) is an independent group with 40 centres from Europe collecting data for collaborative research on ECT. Multiple articles have been published by the group focusing on the outcome and toxicity of ECT of different histological types of tumors including breast cancer,^12^ SCC,^13^ BCC,^14^ MM^15^ and angiosarcoma^16^ The only publication reporting QoL outcomes was the latter one: pain score (VAS) showed a significant decrease 2 months after the treatment, EQ-5D and EQ-VAS scores remained comparable.

Our study is the first one focusing on reporting quality of life outcomes of patients with various histological types of tumors. When evaluating the 3 levels of the EQ-5D questionnaire, we noticed a decrease in the proportion of patients who reported some problems with respect to pain/discomfort and anxiety/depression (Table 2). Overall, we observed a slight, but not statistically significant improvement in EQ-VAS, Pain-VAS and EQ-5D-index values after ECT. In the subgroup analysis, we found that patients who previously underwent radiotherapy had a significant decrease in EQ-VAS scores and a significant increase in EQ-5D index. This somewhat controversial result shows the importance of using multiple QoL questionnaires – one’s overall health state might decrease due to other factors (such as comorbidities or age), and at the same time, they might experience improvement in the five dimensions: mobility, self-care, performing usual activities, pain/discomfort and anxiety/depression. EQ-VAS, Pain-VAS and EQ-5D index both increased significantly after ECT in those who had tumor nodules smaller than 3 cm. Several factors may explain why patients with smaller tumors and those who received prior radiotherapy had better outcomes – in terms of quality of life. Smaller tumors cause less physical and functional impairment, leading to better baseline quality of life. Additionally, we know that smaller tumors generally respond better to electrochemotherapy. A meta-analysis of 1,466 tumors found that tumors smaller than 3 cm had a complete response rate of 59.5% and an objective response rate of 85.7%, while tumors 3 cm or larger had a complete response rate of 33.3% and an objective response rate of 68.2%^17^. Prior radiotherapy may have helped control the disease, reducing distress and improving quality of life. Lastly, patients with smaller tumors or prior treatment may perceive their condition as more manageable, positively affecting their self-reported quality of life. This result shows that this particular subgroup might benefit the most from ECT regarding QoL.

We found that neither the location of the treated tumors nor the number of comorbidities had a statistically significant impact on the evaluated outcomes (EQ-VAS, Pain-VAS, EQ-5D index). Maybe generic QoL instruments such as the EQ-5D may not fully capture nuanced functional or psychosocial impacts related to specific tumor sites or comorbidity profiles. Additionally, the heterogeneity of comorbid conditions within each category may dilute their overall influence on QoL scores.

In an article published in 2015 by Quaglino et al., the authors focused on identifying risk factors for pain after ECT^18^ Their results showed that higher post-ECT pain was significantly associated with pre-treatment pain, larger biggest tumor nodule, previous irradiation and higher treatment current. We found that patients with MM and patients with larger tumor nodules had a slight increase in pain values after ECT. These risk factors for posttreatment pain must be identified prior to treatment, and these patients should have a closer follow-up with appropriate pain management.

Although patients with benign conditions were not included in our cohort, there is growing evidence—including several case reports—supporting the beneficial effects of ECT in non-malignant tumors as well. For instance, Bonadies^19^ et al. described a patient with Brooke-Spiegler syndrome, in whom extensive surgery would have been required to remove multiple scalp cylindromas. Instead, ECT proved effective, contributing to tumor control and improved quality of life. This suggests a potential future role for ECT in the management of selected benign conditions.

Our study has some limitations, including a relatively small sample size and a wide variation in patient age and tumor histological types. Additionally, we did not account for patient comorbidities, which significantly impact changes in their quality of life over time.

## Conclusions

Our results indicate that ECT can maintain, or in some cases improve the patients’ QoL in the short term, highlighting its role in supportive care.

## Data Availability

The datasets used and/or analysed during the current study available from the corresponding author on reasonable request.
